# Microstructured Finger-Joint Interfaces for Reducing Thermal Contact Resistance in Thermal Pad Assemblies

**DOI:** 10.3390/mi17101142

**Published:** 2026-09-30

**Authors:** Menglin Li, Jie Liu, Mingyang Ma

**Affiliations:** 1 State Key Laboratory of Low-Carbon Smart Coal-Fired Power Generation and Ultra-Clean Emission, School of Energy and Environment, Southeast University, Nanjing 210096, China; 2 Key Laboratory of Energy Thermal Conversion and Control of Ministry of Education, School of Energy and Environment, Southeast University, Nanjing, 210096, China

**Keywords:** mechanical contact, thermal interface, electronic thermal management

## Abstract

Thermal pads are widely used for heat dissipation in electronic devices, but their performance can be limited by incomplete contact with the adjoining surfaces. In this study, a double-sided finger-joint interface was introduced to improve thermal contact without modifying the composition of the thermal pad. Two commercial thermal pads were assembled between either planar or finger-structured brass substrates and tested under pressures ranging from 0.1 to 1.0 MPa. For both pads, the finger-joint interface reduced the thermal contact resistance to approximately one-third of that measured with the corresponding planar interface. Surface morphology characterization and mechanical analysis indicated that the finger geometry increased the local normal contact force and promoted more effective contact between the pads and substrates. In a bolt-clamped LED assembly, the finger-joint interface reduced the chip temperature by 49.6 °C at 1.0 A, corresponding to a 45.3% reduction in chip temperature rise. This improvement was retained after 100 heating and cooling cycles. These findings demonstrate that surface geometry provides a practical approach for improving the effective thermal contact performance of thermal pads.

## 1. Introduction

Heat transfer across mechanically joined solids is governed not only by the thermal properties of the constituent materials but also by the quality of contact established at the interface. At a nominally flat joint, real contact occurs only at discrete surface asperities, and the resulting microcontacts occupy only a small fraction of the nominal contact area. Heat flow is therefore constricted through these limited contact spots, producing thermal constriction resistance [1,2,3,4,5]. Increasing the applied load generally enlarges the real contact area and improves interfacial heat transfer. In practical assemblies, however, the allowable load is often constrained by component strength, dimensional stability, and long-term structural reliability [6,7,8,9]. Consequently, achieving low thermal contact resistance under a limited applied load remains an important challenge in the thermal management of mechanically assembled systems.

Electrically insulating thermal pads are widely employed in electronic packaging to couple heat-generating components to heat spreaders, heat sinks, or cold plates while simultaneously providing gap accommodation and electrical isolation [10,11,12,13,14]. These pads are generally composed of a compliant polymer matrix filled with thermally conductive but electrically insulating particles, such as alumina, aluminum nitride, or boron nitride. Their thermal performance is commonly improved by increasing the filler content, constructing continuous heat-conduction networks, controlling filler orientation, introducing hybrid fillers, or modifying the polymer matrix to improve compliance and surface conformity [15,16,17,18,19,20,21]. However, heat passing through an assembled thermal pad interface must cross not only the pad itself but also the two mechanically formed pad–substrate contacts. Accordingly, the total thermal resistance consists of the bulk thermal resistance of the pad and the thermal contact resistances at its two external surfaces [22,23,24]. Limited real contact area can cause these contact resistances to remain substantial, preventing the intrinsic thermal performance of the pad from being fully realized in the assembled interface. Nevertheless, most existing approaches focus on modifying the composition or internal structure of the thermal pad, whereas the geometry of the mating surfaces is generally treated as a fixed boundary rather than as an independent parameter for improving thermal contact.

Surface microstructures have been widely used to control mechanical contact in different functional interfaces. In flexible sensors, microstructured or interlocked interfaces have been used to enhance pressure sensitivity by controlling deformation and the contact area between functional layers. For example, Mannsfeld et al. [25] introduced microstructured dielectric layers, while Bai et al. [26] designed graded interlocked ionic/electrode interfaces to improve sensitivity and linearity. In photovoltaic devices, surface microstructures have also been employed to optimize front-side electrical contacts. Bullock et al. [27] used microchannels to form narrow, high-aspect-ratio metal contacts, whereas Khanna et al. [28] demonstrated that random-pyramid textures affect Ag contact formation and contact resistance in crystalline silicon solar cells. These studies show that surface geometry can be deliberately designed to control contact and the associated transport processes. More recently, Hao et al. [29] reported a carpentry-inspired finger-joint interface for thermal applications and demonstrated that geometric control of mechanical contact can substantially improve interfacial heat transfer. However, that enhancement was achieved using electrically conductive indium as the interfacial material. Plastic deformation of indium, shear-assisted disruption of surface oxides, and localized metal bonding all contributed to the reduction in thermal contact resistance. Commercial electrically insulating thermal pads are polymer-based composites and do not provide these metal-specific contact mechanisms. It therefore remains unclear whether a finger-joint interface can reduce the thermal contact resistance of electrically insulating thermal pads.

In this work, we construct double-sided finger-joint interfaces using two commercial electrically insulating thermal pads with different thicknesses and through-thickness thermal conductivities. Identical pads are assembled between either planar or finger-structured brass substrates and tested over an applied-pressure range of 0.1–1.0 MPa, allowing the effect of surface geometry on thermal contact to be evaluated. The thermal contact resistance at the two pad–brass interfaces is estimated by subtracting the bulk thermal resistance of the pad from the measured thermal resistance of the brass–pad–brass assembly. For both pads, the ratio of the planar-interface contact resistance to the finger-joint-interface contact resistance remains approximately 2.7–3.2 over the investigated pressure range. Thus, the finger-joint interface reduces the thermal contact resistance to approximately one-third of that of the corresponding planar interface. Characterization of the indentations on the thermal pad, reconstruction of the actual finger profiles, and contact-mechanics analysis show that the measured improvement is consistent with amplification of the local normal contact force by the finger geometry. Tests using a bolt-clamped LED assembly further demonstrate a maximum chip-temperature reduction of approximately 50 °C, and this thermal advantage is maintained after 100 heating cycles. These results demonstrate that the geometry of the mating surfaces can be designed to improve the effective thermal performance of commercial electrically insulating thermal pads without modifying their material composition.

## 2. Materials and Methods

### 2.1. Thermal Pad Materials and Thermophysical Properties

Two commercially available thermal pads were investigated. Thermal pad #1 was Model 8W supplied by Beilong Electronics Co., Ltd. (Guangzhou, China) and thermal pad #2 was Bergquist (Chanhassen, MN, USA) SIL-PAD 2000. The original thicknesses of pads #1 and #2 were 0.29 and 0.27 mm, respectively. Here, “8W” denotes the product model of pad #1 rather than its thermal conductivity.

The specific heat capacity, *C*_p_, of each thermal pad was measured by differential scanning calorimetry (DSC), and the through-thickness thermal diffusivity, *α*, was measured by laser flash analysis (LFA). The through-thickness thermal conductivity of the thermal pad, *k*_TIM_, was calculated as

(1) $$k_{\mathrm{TIM}}\;=\alpha \times \;\rho \times C_{p}\;$$

where *ρ* is the density of the thermal pad. The measured thermal conductivities of pads #1 and #2 were 2.47 and 3.34 W m^−1^ K^−1^, respectively.

The area-normalized bulk thermal resistance of each thermal pad was estimated from

(2) $$R_{\mathrm{bulk}}\;=\frac{t_{0}}{k_{\mathrm{TIM}}}\;\;\;$$

where *t*_0_ is the original thickness of the thermal pad. The resulting *R*_bulk_ values were 117.6 and 80.7 K mm^2^ W^−1^ for pads #1 and #2, respectively. The material properties used in the analysis are summarized in Table 1.

Values are reported as mean ± standard deviation where repeated measurements were available. Thermal diffusivity was measured three times using laser-flash analysis, and thickness was measured at four positions along two orthogonal directions. The reported variability in density accounts for repeated mass and dimensional measurements. Specific heat capacity was obtained from a single differential scanning calorimetry measurement and is therefore reported without a repeatability-based standard deviation. The uncertainties in through-thickness thermal conductivity and *R*_bulk_ were obtained by propagation of the available measurement variability.

### 2.2. Design and Assembly of the Double-Sided Finger-Joint Interface

**Figure 1:**
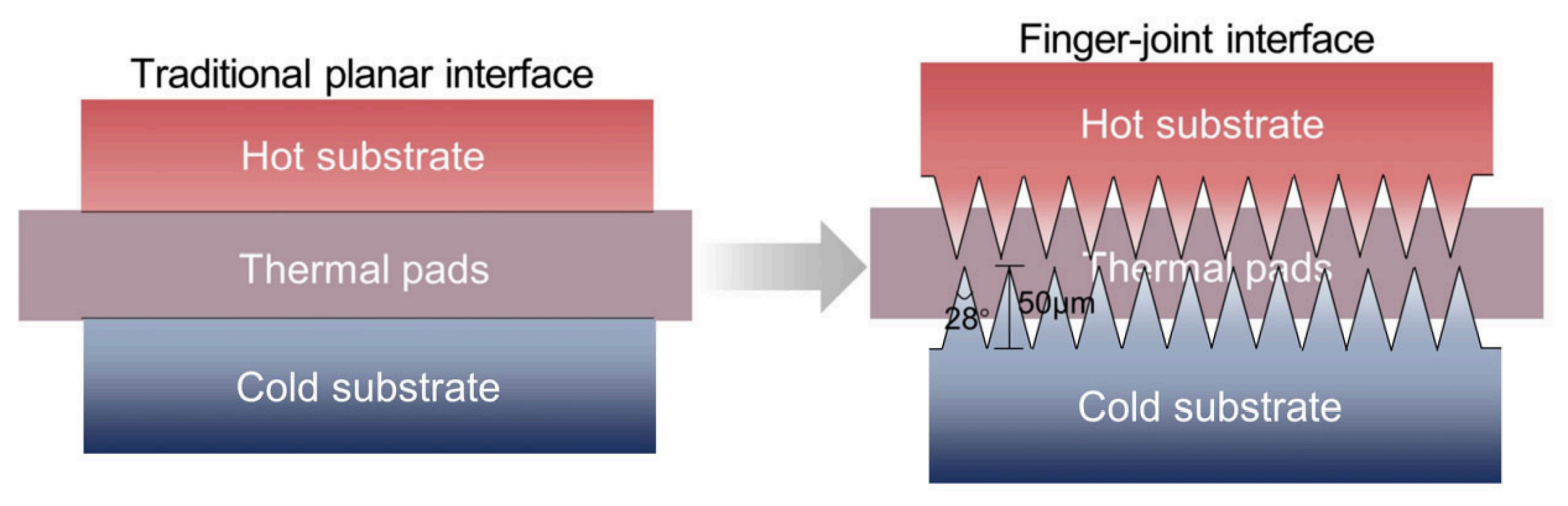
Schematic comparison of the planar and double-sided finger-joint assemblies. In the planar assembly, the thermal pad is clamped between two flat brass substrates. In the finger-joint assembly, the pad is clamped between two brass substrates bearing concentric finger arrays with a nominal height of 50 μm and a nominal apex angle of 28°.

Planar and finger-joint assemblies were prepared to evaluate the effect of substrate surface geometry on the thermal resistance of the thermal pad interfaces (Figure 1). In the planar assembly, a thermal pad was clamped between two brass substrates with nominally flat opposing surfaces. In the finger-joint assembly, the same type of thermal pad was clamped between two brass substrates containing pointed finger arrays on their opposing surfaces. The pad material, apparent surface area, and applied pressure were kept the same for the planar and finger-joint assemblies.

The finger arrays were designed as densely spaced concentric circular ridges. Each ideal triangular finger had a nominal design height of 50 μm and a nominal apex angle of approximately 28°. These values describe the nominal design geometry rather than the as-fabricated profile. During assembly, the thermal pad was placed between the upper and lower finger-structured substrates, and a normal load was applied to compress the complete brass–pad–brass stack. No deliberate peak-to-valley or peak-to-peak registration was imposed between the upper and lower finger arrays. Therefore, the measured thermal resistance represents the overall response of the numerous finger–pad contacts distributed across both sides of the thermal pad rather than the response of individually aligned pairs of opposing fingers.

The finger-array geometry was selected as a mechanism-driven proof-of-concept design rather than through formal numerical optimization. The concentric-ring arrangement was adopted primarily because it is well suited to fabrication by lathe turning. Rotation of the brass substrate enables the cutting tool to form continuous annular ridges and valleys through controlled radial positioning, providing a practical means of producing a dense finger array over the contact surface. In the nominal design, each ideal triangular finger had a height of 50 μm and a base width of 25 μm. Because adjacent triangular fingers were contiguous, the base width was also equal to the peak-to-peak period. These dimensions were selected to provide an acute apex angle and a dense distribution of contact features while remaining compatible with the available turning process.

### 2.3. Measurement of Thermal Resistance

**Figure 2:**
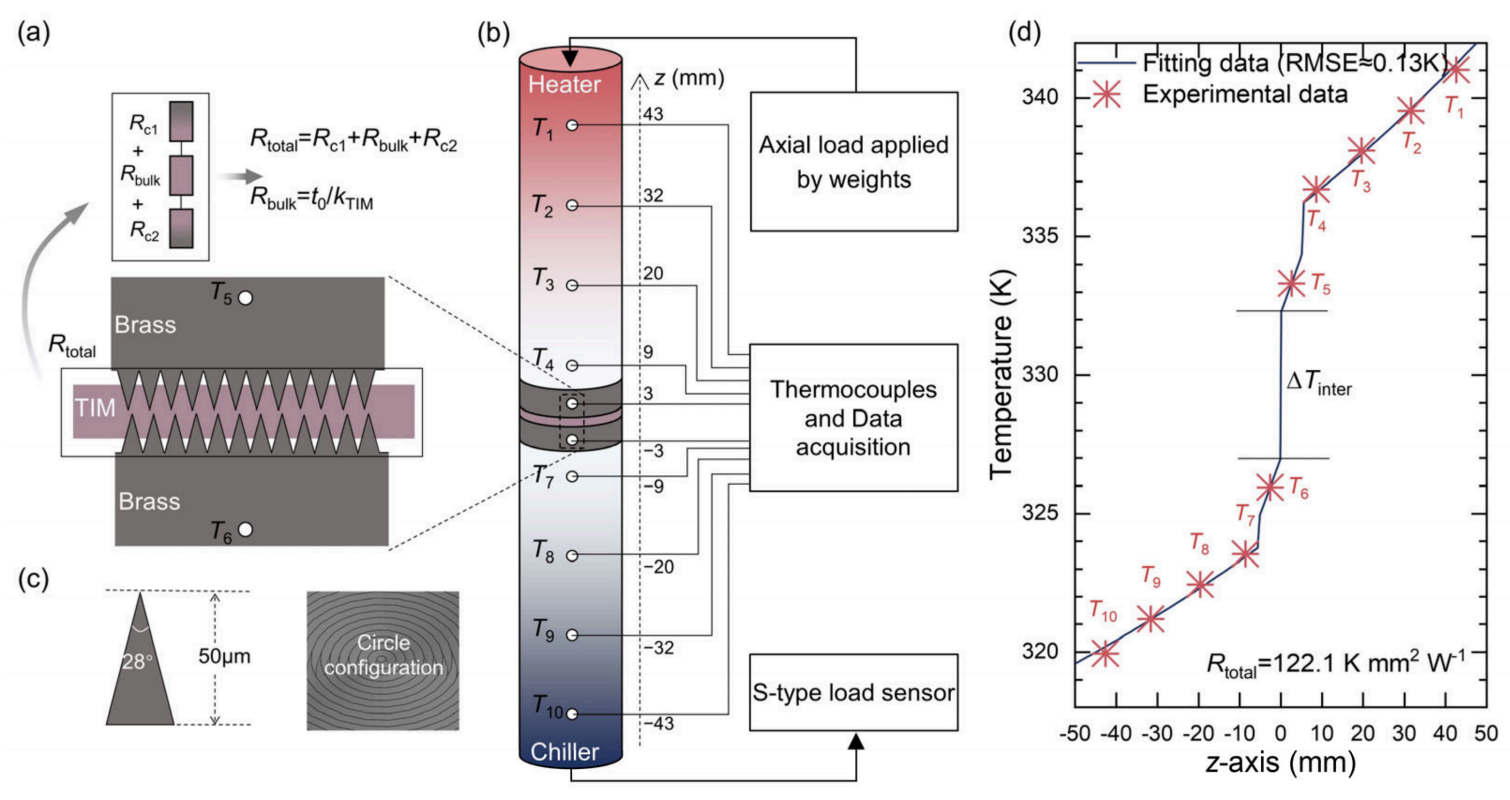
Steady-state measurement of the thermal resistance of the brass–pad–brass assemblies. (**a**) Thermal-resistance network of the assembly; (**b**) schematic of the measurement apparatus; (**c**) nominal geometry design and concentric arrangement of the brass finger structures; (**d**) Comparison between the measured temperatures and the temperature distribution obtained from the optimized finite element model. In (**d**), the symbols represent the directly measured temperatures at *T*_1_–*T*_10_, whereas the solid line represents the fitted finite-element temperature profile. *T*_5_ and *T*_6_ were located within the brass substrates rather than directly at the brass–pad interfaces, and the thermal resistances of the intervening brass segments were included in the analysis.

The thermal resistance of each brass–pad–brass assembly was measured using a custom steady-state apparatus based on ASTM D5470 (Figure 2a–c) [30]. The sample assembly was placed between two oxygen-free high-conductivity copper reference bars. A heater attached to the top of upper reference bar supplied heat, while the bottom of lower reference bar was connected to a chiller, establishing predominantly axial heat flow through the sample. The normal load was applied using calibrated weights and monitored by an S-type load cell positioned beneath the lower reference bar. The applied force was divided by the nominal interface area to obtain the applied pressure, which was varied from 0.1 to 1.0 MPa.

Temperatures *T*_1_–*T*_4_ and *T*_7_–*T*_10_ were measured using thermocouples installed at prescribed axial positions in the upper and lower copper reference bars. Two additional thermocouples, *T*_5_ and *T*_6_, were inserted into the brass substrates immediately above and below the interface. Because the temperature-dependent thermal conductivity and dimensions of the brass substrates, together with the exact distances from the thermocouple junctions to the interfaces, were known, the area-normalized bulk thermal resistances of the intervening brass segments could be calculated and incorporated into the finite-element model. The temperature drops across these brass segments could therefore be excluded, allowing *R*_total_ to be determined from *T*_5_ and *T*_6_ together with the heat flux constrained by the reference-bar measurements. A thermocouple was not placed directly inside the 0.27–0.29 mm thick compliant pad because its insertion would alter the local deformation, contact pressure, and heat-flow path.

The measured area-normalized total thermal resistance of the brass–pad–brass assembly, *R*_total_, consists of the bulk thermal resistance of the pad and the thermal contact resistances at its upper and lower interfaces (Figure 2a):

(3) $$R_{\mathrm{total}}\;=R_{c,\mathrm{upper}}+R_{\mathrm{bulk}}\;+R_{c,\mathrm{lower}}\;$$

where *R*_c,upper_ and *R*_c,lower_ are the area-normalized thermal contact resistances at the two pad–brass interfaces. The total thermal contact resistance at the two interfaces, *R*_c_, was estimated as

(4) $$R_{c\;}=R_{c,\mathrm{upper}}\;+R_{c,\mathrm{lower}}\;=R_{\mathrm{total}\;}-R_{\mathrm{bulk}}\;$$

All thermal resistance values reported in this study are expressed in K mm^2^ W^−1^.

Because heat losses from the exposed surfaces could cause the heat flow in the reference bars to deviate from an ideal one-dimensional condition, finite element simulations were used to reproduce the complete temperature distribution of the apparatus. Natural convection and surface radiation were included in the model. The total thermal resistance of the sample assembly, the heat input at the upper boundary, the heat removed at the lower boundary, and the contact resistances between the brass substrates and copper reference bars were iteratively adjusted using MATLAB (2020). The optimized parameters were obtained by minimizing the root-mean-square error (RMSE) between the measured and simulated temperatures:

(5) $$RMSE=\sqrt{\frac{1}{N}\sum_{i=1}^{n}{\left(T_{i,\mathrm{sim}}\;-T_{i,\exp}\right)}^{2}}\;$$

where (*N* = 10) is the number of temperature measurements. As shown in Figure 2d, the simulated temperature distribution closely reproduces the experimental data for an example fitting result, with an RMSE of 0.13 K.

### 2.4. Device-Level LED

A device-level LED test was performed to evaluate the thermal performance of the planar and finger-joint assemblies under practical conditions. The LED package was thermally coupled to a copper heat sink through either a planar interface or a double-sided finger-joint interface. The two assemblies used the same LED package, thermal pad #2, heat sink, and nominal interface area, with the surface geometry of the clamped interface being the only design difference.

The assembly was secured using four bolts, producing an estimated nominal pressure of approximately 0.31 MPa. The LED was powered by a direct-current supply and operated sequentially at currents of 0.7 and 1.0 A. Thermocouples were used to monitor the LED chip temperature, *T*_chip_, the heat-sink temperature, *T*_heat,sink_, and the ambient temperature, *T*_amb_. The temperatures were continuously recorded during heating and subsequent cooling.

The planar and finger-joint assemblies were subsequently subjected to 100 heating and cooling cycles under the same clamping condition. After cycling, their temperature responses were measured again using the same current sequence. The initial and post-cycling temperature responses were compared to evaluate the stability of the thermal interfaces under repeated operation.

### 2.5. Characterization of the Finger–Pad Contact Geometry

Thermal pad #2 was used to characterize the local contact formed between the thermal pad and the brass fingers. The pad was compressed between the two finger-structured brass substrates at an applied pressure of 1.0 MPa. After unloading, the pad was removed and its surfaces were examined using a microscope. The widths of multiple clearly resolved indentations left by the brass fingers were measured from the microscopic images using ImageJ (version 1.53e) and averaged to obtain the characteristic indentation width, *w*_avg_. This width was used as an experimental estimate of the lateral extent of the finger–pad contact.

The surface topography of the finger-structured brass substrate before loading was measured using a surface profilometer. Profiles passing across multiple finger peaks were extracted from the measured topography. Because the machined peaks deviated from the nominal triangular geometry, a horizontal reference plane was introduced to identify the portion of each peak that participated in contact. For a given reference-plane height, the two intersections between the plane and the *i*-th peak profile defined a local width *d_i_*. The reference-plane height was adjusted until the mean width of the selected peak regions was equal to the measured average indentation width:

(6) $$w_{\mathrm{avg}}\;=\frac{1}{n}\sum_{i=1}^{n}d_{i}\;$$

where *n* is the number of finger peaks included in the analysis.

The portion of each peak above the selected reference plane was then extracted. To compare peaks having different widths and heights, the lateral coordinate was normalized by *d_i_*, and the height was referenced to the selected plane and normalized by the same width. The normalized peak profiles were subsequently averaged to obtain a representative contact profile. The effective peak angle was determined from the slopes of the two sides of this averaged profile and was used in the subsequent contact-mechanics analysis.

## 3. Results

### 3.1. Thermal Resistance of the Planar and Finger-Joint Assemblies

**Figure 3:**
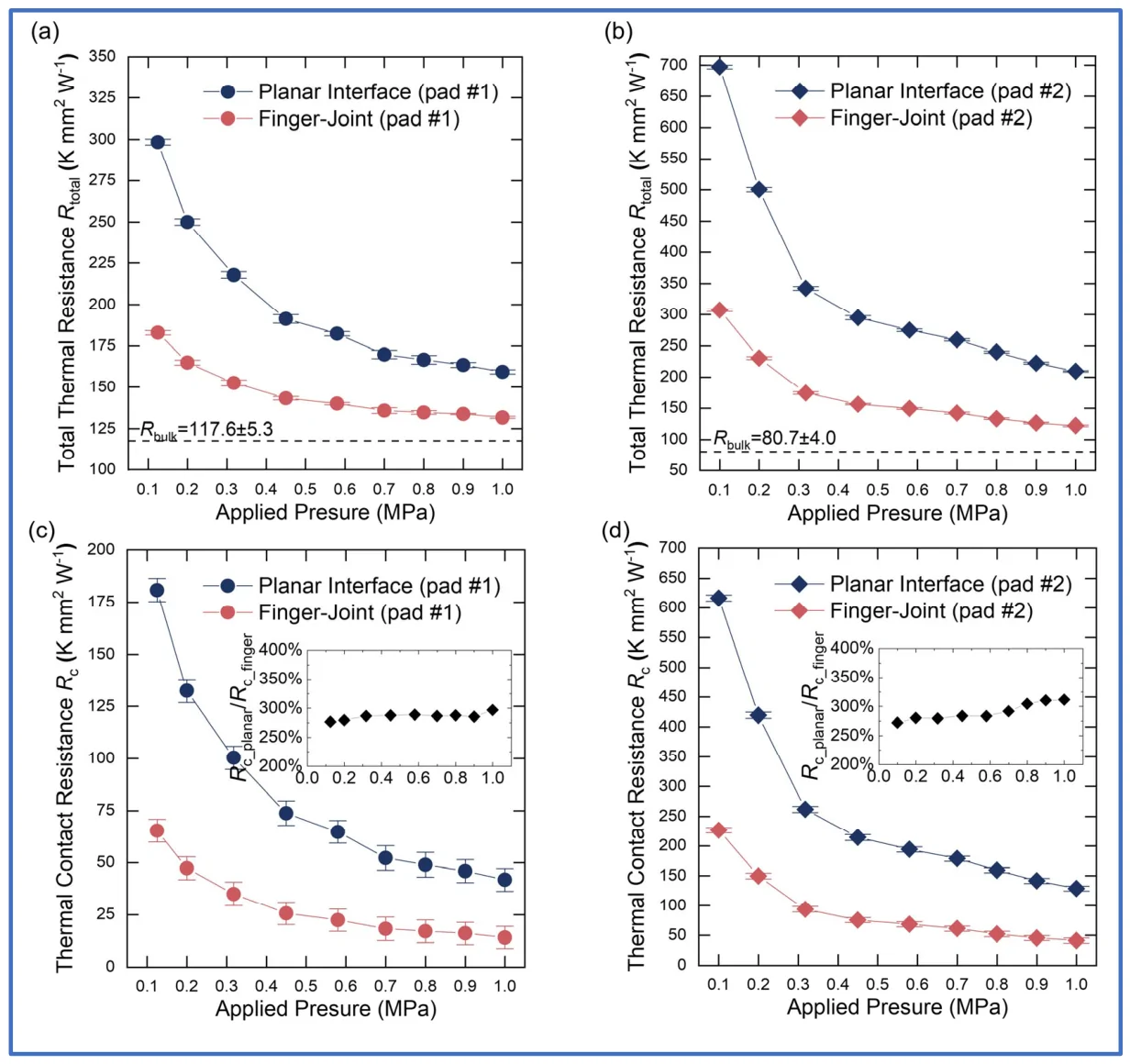
Pressure-dependent thermal resistance of the planar and finger-joint assemblies. (**a**) Total thermal resistance, *R*_total_, of assemblies containing thermal pad #1; (**b**) *R*_total_ of assemblies containing thermal pad #2; (**c**) total thermal contact resistance, *R*_c_, at the two pad–brass interfaces for pad #1; (**d**) *R*_c_ for pad #2. The dashed lines in (**a**,**b**) indicate the estimated bulk thermal resistances of the pads. The insets in (**c**,**d**) show the ratio *R*_c,planar_/*R*_c,finger_. Each data point in (**a**,**b**) represents the mean of five independently fitted *R*_total_ values, and the error bars indicate one standard deviation (*s_R_*_total_). The values of *R*_c_ in (**c**,**d**) were calculated as *R*_c_ = *R*_total_ − *R*_bulk_, and their error bars represent the propagated standard deviations of *R*_total_ and *R*_bulk_: *s_R_*_c_ = (*s*^2^*_R_*_total_ + *s*^2^*_R_*_bulk_)^1/2^.

For both thermal pads, *R*_total_ decreased with increasing pressure and was consistently lower in the finger-joint assembly than in the planar assembly (Figure 3a,b). For pad #1, increasing the pressure from 0.1 to 1.0 MPa reduced *R*_total_ from approximately 298 to 158 K mm^2^ W^−1^ in the planar assembly and from approximately 181 to 130 K mm^2^ W^−1^ in the finger-joint assembly. At 1.0 MPa, the latter value approached the estimated bulk resistance of pad #1 (*R*_bulk_ = 117.6 K mm^2^ W^−1^), indicating that the remaining contribution from contact resistance was relatively small. For pad #2, *R*_total_ decreased from approximately 690 to 205 K mm^2^ W^−1^ in the planar assembly and from approximately 305 to 122 K mm^2^ W^−1^ in the finger-joint assembly over the same pressure range.

To isolate the contact-resistance contribution, the estimated bulk resistance of each pad was subtracted from *R*_total_ according to Equation (4). The resulting thermal contact resistance, *R*_c_, at the two pad–brass interfaces is shown in Figure 3c,d. For pad #1, *R*_c_ at 0.1 MPa was approximately 180 K mm^2^ W^−1^ for the planar interface and approximately 64 K mm^2^ W^−1^ for the finger-joint interface. At 1.0 MPa, the corresponding values decreased to approximately 40 and 12 K mm^2^ W^−1^, respectively. For pad #2, *R*_c_ decreased from approximately 610 to 125 K mm^2^ W^−1^ for the planar interface and from approximately 225 to 40 K mm^2^ W^−1^ for the finger-joint interface as the pressure increased from 0.1 to 1.0 MPa.

The insets of Figure 3c,d show the ratio *R*_c,planar_/*R*_c,finger_. Despite the substantial differences in the absolute contact resistances of the two pads, this ratio remained within approximately 2.7–3.2 over the investigated pressure range. Thus, the double-sided finger-joint structure reduced the thermal contact resistance at the two pad–brass interfaces to approximately one-third of that of the corresponding planar interface. The similar resistance ratios obtained for the two commercial pads suggest that the relative improvement is primarily associated with the finger geometry. The physical origin of this approximately threefold improvement is examined in Section 4.1.

For each thermal pad material, two separate specimens cut from the same commercial pad material were used for the planar and finger-joint configurations. The applied pressure was increased monotonically over the investigated range without precompression, unloading, or reassembly between successive pressure levels. After each pressure adjustment, the assembly was allowed to reach steady state for approximately 30 min. Five complete temperature datasets, each comprising readings at *T*_1_–*T*_10_, were subsequently recorded at 1 min intervals. Each dataset was separately fitted using the finite-element model, yielding five *R*_total_ values at each pressure. The *R*_total_ values in Figure 3a,b are therefore reported as the mean ± one sample standard deviation (*n* = 5). The corresponding *R*_c_ values in Figure 3c,d were calculated as *R*_c_ = *R*_total_ − *R*_bulk_, with their standard deviations obtained by propagating the uncertainties in *R*_total_ and *R*_bulk_ according to *s_R_*_c_ = (*s*^2^*_R_*_total_ + *s*^2^*_R_*_bulk_)^1/2^. The 30 min holding period at each pressure, together with the relatively small variation among the five subsequent temperature datasets, indicates that sufficient time was allowed for the short-term deformation of the compliant pad, the interfacial contact state, and the thermal response to stabilize before data acquisition. Because the loading protocol did not include an unloading branch, pressure-dependent loading–unloading hysteresis was not quantitatively evaluated in the present study.

### 3.2. Device-Level Thermal Performance

**Figure 4:**
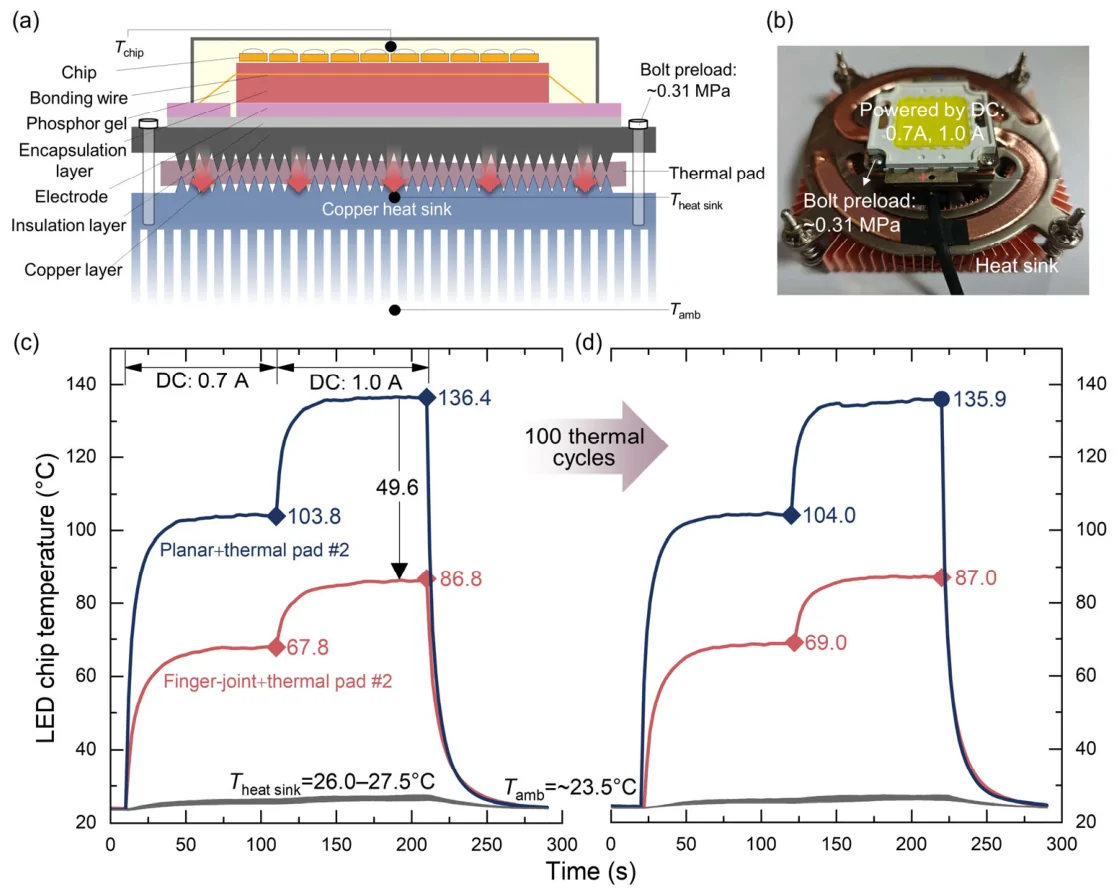
Device-level thermal performance of the planar and finger-joint interfaces containing thermal pad #2. (**a**) Schematic of the bolt-clamped LED assembly and heat-transfer path; (**b**) photograph of the experimental assembly; (**c**) LED chip-temperature responses under sequential currents of 0.7 and 1.0 A before thermal cycling; (**d**) corresponding temperature responses after 100 heating and cooling cycles.

Figure 4a,b show the bolt-clamped LED assembly used to evaluate the planar and finger-joint interfaces under device-level operating conditions. Thermal pad #2 was used in both assemblies, and the nominal clamping pressure was approximately 0.31 MPa. Because the LED assembly involved a relatively large mating area, the four corner bolts were tightened to the same preset torque using a torque wrench to improve the uniformity of the clamping load and help maintain parallel contact across the interface. During the measurements, the ambient temperature remained at approximately 23.5 °C, while the heat-sink temperature remained within approximately 26.0–27.5 °C. The similar heat-sink temperatures allowed the differences in LED chip temperature to be attributed primarily to the thermal resistance of the two interface configurations. The reported chip temperatures represent individual steady-state values obtained from each interface configuration rather than averages from independently repeated assemblies.

The initial temperature responses are shown in Figure 4c. At an applied current of 0.7 A, the chip temperature reached approximately 103.8 °C with the planar interface and 67.8 °C with the finger-joint interface, corresponding to a temperature reduction of 36.0 °C. When the current was increased to 1.0 A, the chip temperatures increased to approximately 136.4 and 86.8 °C for the planar and finger-joint interfaces, respectively. The finger-joint interface therefore reduced the maximum chip temperature by 49.6 °C.

To compare the thermal improvement independently of the temperature of heat sink, the relative reduction *η*_∆T_ in chip temperature rise was calculated as

(7) $$\eta_{\Delta T\;}=\frac{T_{\mathrm{chip},\mathrm{planar}}\;-T_{\mathrm{chip},\mathrm{finger}\;}\;}{T_{\mathrm{chip},\mathrm{planar}}-T_{\mathrm{heat}\;\mathrm{sink}}}$$

The finger-joint interface reduced the chip temperature rise by *η*_∆T_ ≈ 45.3% at 1.0 A. These results demonstrate that the reduction in thermal contact resistance measured using the steady-state apparatus translated directly into a substantial decrease in device operating temperature.

The temperature responses measured after 100 heating and cooling cycles are shown in Figure 4d. At 0.7 A, the chip temperatures were approximately 104.0 °C for the planar interface and 69.0 °C for the finger-joint interface. At 1.0 A, the corresponding temperatures were approximately 135.9 and 87.0 °C. Compared with the initial measurements, the changes in the steady-state temperatures were no greater than approximately 1.2 °C. The temperature difference between the two assemblies remained approximately 35.0 °C at 0.7 A and 48.9 °C at 1.0 A, indicating that the thermal advantage of the finger-joint interface was retained after repeated operation.

## 4. Discussion

### 4.1. Mechanism of Thermal Contact Enhancement

**Figure 5:**
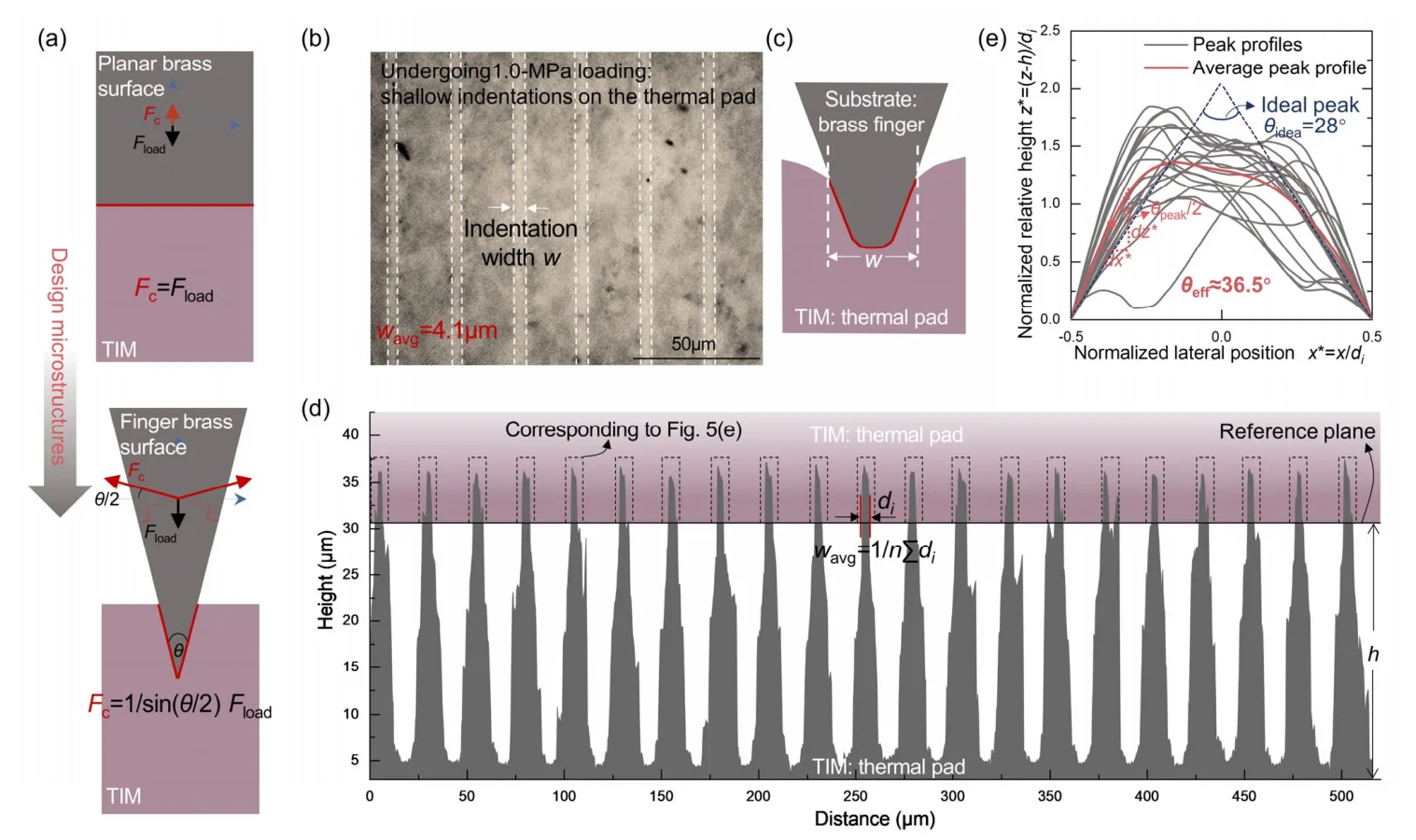
Characterization and analysis of the finger–pad contact. (**a**) Force balance for planar and finger contacts under the applied axial load; (**b**) microscope image of indentations formed on thermal pad #2 after loading at 1.0 MPa; (**c**) schematic of the local contact between a machined brass finger and the thermal pad; (**d**) selection of the contacting regions from the measured finger profiles using the average indentation width; (**e**) normalized finger-peak profiles and their representative average profile used to determine the effective apex angle. In (**d**), the reconstructed as-fabricated finger has a peak-to-valley height of approximately 35 μm. The dashed box indicates the contact-relevant region determined from the indentation on the thermal pad, corresponding to an engagement depth of approximately 5–10 μm. The effective apex angle was evaluated from the finger profile above the resulting contact reference plane.

Although pads #1 and #2 exhibited substantially different absolute thermal contact resistances, their *R*_c,planar_/*R*_c,finger_ ratios were both close to three over the pressure range. This similar relative improvement suggests that the finger geometry, rather than the properties of a particular thermal pad, played the dominant role in determining the reduction in thermal contact resistance. To examine this mechanism, the local contact formed between the brass fingers and thermal pad #2 was characterized after loading at 1.0 MPa (Figure 5).

For a planar interface under an applied normal load *F*_load_, the resultant normal contact force is equal to the applied load (Figure 5a). For an ideal symmetric finger with an apex angle *θ*, force equilibrium in the loading direction gives

(8) $$F_{\mathrm{load}}\;=2F_{s}\;sin(\frac{\theta }{2}\;)$$

where *F*_s_ is the normal force acting on one side of the finger. The total normal contact force acting on the two inclined sides is therefore

(9) $$F_{c}\;=2F_{s\;}=\frac{1}{sin(\frac{\theta }{2})}F_{\mathrm{load}}\;\;$$

Accordingly, the finger geometry converts the applied axial load into a larger total normal force at the inclined finger–pad contacts. Using the nominal apex angle of 28°, Equation (9) gives an ideal force-amplification factor of approximately 4.13. However, this value assumes perfectly sharp and regular finger peaks and therefore represents an ideal geometric limit rather than the force amplification produced by the machined brass surfaces.

To numerically verify the idealized force-amplification mechanism described above, a two-dimensional axisymmetric finite-element contact model was established using the nominal finger geometry, with detailed model settings provided in the Supplementary Materials (Figure S1 and Table S1) [31,32,33]. Under a uniformly applied pressure of 1.0 MPa, the integrated external load was *F*_load_ = 3.14 N, whereas the scalar surface integral of the local normal contact traction over all inclined finger–pad contact surfaces was *F*_c_ = 12.95 N. The resulting ratio, *F*_c_/*F*_load_ = 4.124, agrees closely with the analytical value of 4.13 calculated for the nominal apex angle of 28°, with a difference of approximately 0.2%. Here, *F*_c_ represents the scalar sum of the local normal contact forces acting on the inclined surfaces. The simulation therefore verifies the geometrical force-amplification mechanism illustrated in Figure 5a.

The microscope image in Figure 5b shows the indentations left on thermal pad #2 after 1.0-MPa loading. The indentations had an average width of approximately 4.1 μm, which was much smaller than the nominal width (25.0 μm) of a complete finger. This observation indicates that contact was concentrated near the finger peaks rather than extending over the entire inclined surfaces. The measured indentation width was therefore used to identify the portions of the actual brass profiles that were most likely involved in contact.

Although the nominal design height of each finger was 50 μm, the reconstructed as-fabricated profiles in Figure 5d exhibited a peak-to-valley height of approximately 35 μm. This difference arose because the finite nose radius of the cutting tool produced rounded valley roots that could not reproduce the ideally sharp triangular valleys. As shown in Figure 5d, a reference plane was positioned across the measured finger profiles such that the average width of the profile sections above the plane was equal to the measured indentation width. The vertical distance from this plane to the finger peaks, corresponding to the actual engagement depth, was approximately 5–10 μm, as indicated by the dashed box. This depth was substantially smaller than the measured finger height, indicating that the thermal pad contacted only the upper portion of the fingers and remained well above the valley region. Therefore, the difference between the nominal and measured overall finger heights was considered negligible for the actual contact state under the investigated loading conditions. The selected peak sections above the reference plane were then normalized and averaged to obtain the representative contact profile shown in Figure 5e. Because the local included angle of an as-fabricated peak varies with engagement depth, the effective apex angle was evaluated from this contact-relevant profile rather than from the complete peak-to-valley geometry. The resulting effective apex angle, *θ*_eff_, was approximately 36.5°, giving a contact-force amplification factor of

(10) $$A_{F}\;=\;\frac{F_{c}}{F_{\mathrm{load}}}\;\;=\frac{1}{sin(\frac{\theta_{\mathrm{eff}}}{2})}$$

The difference between the nominal and effective apex angles mainly originates from the finite radius of the machining tool, which modifies the local apex and valley geometries and produces a smoother profile than the ideal geometry. Compared with the ideal amplification factor of approximately 4.13 for the nominal 28° apex angle, the factor of approximately 3.0 for the reconstructed effective angle of 36.5° represents a reduction of approximately 27.4%. Nevertheless, a substantial geometrical amplification is retained. Thus, variations in the as-fabricated apex angle affect the quantitative magnitude of the predicted amplification but do not eliminate the underlying contact-enhancement mechanism. The classical Cooper–Mikic–Yovanovich thermal contact model relates thermal contact conductance to contact pressure through an approximately power-law dependence [34,35,36]. When the material and surface parameters are otherwise treated as unchanged, the conductance scales approximately as

(11) $$h_{c\;}\propto {P_{c}}^{0.95}\;$$

where *P*_c_ is the effective contact pressure. Applying the geometric force-amplification factor from Equation (10) gives

(12) $$\frac{h_{c,\mathrm{finger}}}{h_{c,\mathrm{planar}}}\;\;={\left(\frac{F_{c,\mathrm{finger}}}{F_{c,\mathrm{planar}}}\right)}^{0.95}\;={A_{F}}^{0.95}$$

Because thermal contact conductance is the reciprocal of thermal contact resistance for the same nominal interface area,

(13) $$\frac{h_{c,\mathrm{finger}}}{h_{c,\mathrm{planar}}}\;\;=\frac{R_{c,\mathrm{planar}}}{R_{c,\mathrm{finger}}}\;\;\;\;$$

The predicted conductance ratio of approximately 3.01 agrees with the measured resistance ratios of approximately 2.7–3.2 for both thermal pads. This agreement indicates that amplification of the local normal contact force by the actual finger geometry accounts for the main reduction in thermal contact resistance.

### 4.2. Influence of Thermal Pad Properties

The two thermal pads exhibited different bulk and contact resistances. Pad #2 had a higher measured through-thickness thermal conductivity and a lower estimated bulk resistance than pad #1. Nevertheless, its planar assembly exhibited a substantially higher total thermal resistance, particularly at low pressure. For example, at 0.1 MPa, the estimated bulk resistance of pad #2 was only 80.7 K mm^2^ W^−1^, whereas the measured total resistance of the planar assembly was approximately 690 K mm^2^ W^−1^. The majority of the measured resistance therefore originated from the two pad–brass contacts rather than from heat conduction through the pad itself. This comparison shows that a lower bulk resistance does not necessarily result in a lower resistance after assembly, and poor external contact can mask the benefit of a relatively low bulk resistance.

After introduction of the finger-joint structure, the total resistance of the pad #2 assembly decreased more substantially and approached that of the pad #1 finger-joint assembly at high pressure. Thus, reducing the contact-resistance contribution allowed the lower bulk resistance of pad #2 to contribute more effectively to the overall thermal performance. This result highlights the need to consider the thermal pad and the geometry of the contacting surfaces as a complete assembly rather than evaluating a thermal pad solely from its intrinsic thermal conductivity. Moreover, despite their different absolute resistance values, both pads exhibited similar *R*_c,planar_/*R*_c,finger_ ratios. This consistency supports the interpretation that the relative improvement was governed mainly by the finger geometry.

### 4.3. Practical Implications

The upper and lower finger arrays were assembled without deliberate peak-to-valley or peak-to-peak registration. The observed reduction in thermal resistance was therefore achieved under a relatively simple assembly condition and did not depend on precise matching between individual opposing fingers. Because the interface contained a large number of concentric finger structures, the measured resistance represents the average response of the distributed finger–pad contacts. This tolerance to uncontrolled relative positioning is beneficial for practical assembly. The distributed-contact configuration is also expected to provide some tolerance to moderate local variations in peak height, apex shape, and valley geometry because the overall interfacial conductance arises from the accumulated contributions of numerous finger–pad contacts. The ideal wedge prediction should therefore be regarded as a mechanistic estimate, with the actual force amplification also affected by the local apex shape, tip rounding, friction, and deformation of the compliant thermal pad.

The device-level LED test further demonstrates the practical effect of the reduced contact resistance. The improvement was retained after 100 heating and cooling cycles. These results show that the contact-resistance reduction measured using the reference-bar apparatus can be translated into a substantial reduction in device operating temperature under bolt-clamped conditions.

## 5. Conclusions

This study demonstrates that contact surface geometry can improve the effective thermal performance of commercial electrically insulating thermal pads. In the planar assemblies, a lower thermal bulk resistance did not necessarily produce a lower total resistance, highlighting the dominant contribution of the pad–substrate contacts. The double-sided finger-joint structure suppressed this contribution and enabled the intrinsic thermal properties of the pads to be utilized more effectively.

The observed indentation patterns and reconstructed finger profiles indicate that contact was concentrated near the finger peaks. Contact-mechanics analysis showed that the inclined surfaces amplified the local normal contact force. The amplification predicted from the effective apex angle was consistent with the measured reduction in thermal contact resistance, supporting the proposed geometry-driven mechanism.

The device-level experiment further confirmed that this reduction in interfacial resistance translated into a substantially lower operating temperature and remained effective after repeated operation. Overall, the results establish contact surface geometry as an effective design strategy for enhancing the thermal performance of thermal pad assemblies without modifying the pad material itself.

## Figures and Tables

**Table 1:** Thermophysical properties of the two thermal pads.

Thermal Pad	Manufacturer and Product	Thickness (mm)	Density (g cm^−3^)	Specific Heat Capacity (J g^−1^ K^−1^)	Thermal Diffusivity (mm^2^ s^−1^)	Through-Thickness Thermal Conductivity (W m^−1^ K^−1^)	*R*_bulk_ (K mm^2^ W^−1^)
Pad #1	Beilong Electronics Co., Ltd.; Model 8W	0.29 ± 0.01	3.50 ± 0.02	0.99	0.71 ± 0.02	2.47 ± 0.07	117.6 ± 5.3
Pad #2	Bergquist; SIL-PAD 2000	0.27 ± 0.01	1.94 ± 0.01	1.13	1.52 ± 0.05	3.34 ± 0.11	80.7 ± 4.0

## Data Availability

The original contributions presented in this study are included in the article. Further inquiries can be directed to the corresponding author.

## Supplementary Materials

The following supporting information can be downloaded at: https://www.mdpi.com/article/10.3390/mi17101142/s1, Figure S1: Axisymmetric finite-element verification of the force-amplification mechanism of the ideal finger array. (a) Model geometry and mechanical boundary conditions. The bottom surface of the thermal pad was fixed, and a uniform pressure of 1.0 MPa was applied to the upper surface of the brass substrate. (b) Contact-pressure distribution at the finger–pad interface; Table S1: Comparison of the boundary conditions and system settings used in the finite-element model and the experiment.
