# Supplementary material for: Microstructured Finger-Joint Interfaces for Reducing Thermal Contact Resistance in Thermal Pad Assemblies
Source: Micromachines (Basel). 2026 Sep 30;17(10):1142. doi: 10.3390/mi17101142 (PMC13706979; doi:10.3390/mi17101142)
Supplement: Supplementary file 1 [file micromachines-17-01142-s001.zip › micromachines-4548024-supplementary.pdf]

Finite-Element Verification of the Ideal Finger-Array Force Amplification

A two-dimensional axisymmetric finite-element model was established to verify the force-amplification mechanism of the ideal finger-array geometry. The model consisted of an upper brass substrate containing contiguous, ideally sharp triangular fingers and a lower thermal pad. Each nominal finger had a height of 50 μm and a base width of 25 μm. Because the adjacent triangular fingers were contiguous, the base width was also equal to the peak-to-peak period. These dimensions corresponded to a nominal apex angle of ~28°.

The brass substrate was represented as an isotropic linear-elastic material, whereas the thermal pad was described using an isotropic elastic–plastic model to account for the local irreversible deformation produced beneath the finger tips. Representative effective elastic properties were adopted, with Young's modulus and Poisson's ratio set to approximately 105 GPa and 0.34 for brass and approximately 20 MPa and 0.49 for the thermal pad, respectively [1-3]. The two bodies were constructed as an assembly and coupled through a contact pair, allowing nonmatching meshes on the opposing contact boundaries. The bottom surface of the thermal pad was fixed, the central axis was assigned an axisymmetric boundary condition, and a uniform compressive pressure of 1.0 MPa was applied to the upper surface of the brass substrate.

The externally applied force, F_{load}, was obtained by integrating the applied pressure over the upper loading surface. The amplified contact force, Fc, was calculated as the scalar surface integral of the local normal contact traction over all inclined finger–pad contact surfaces. The axisymmetric $2\pi r$ weighting was included in both integrations. The calculated values were F_{load}=3.14 N and F_c=12.95 N, giving F_c/F_{load}=4.124. This result agrees closely with the theoretical value of 4.133 predicted by the ideal wedge model for an apex angle of 28°.

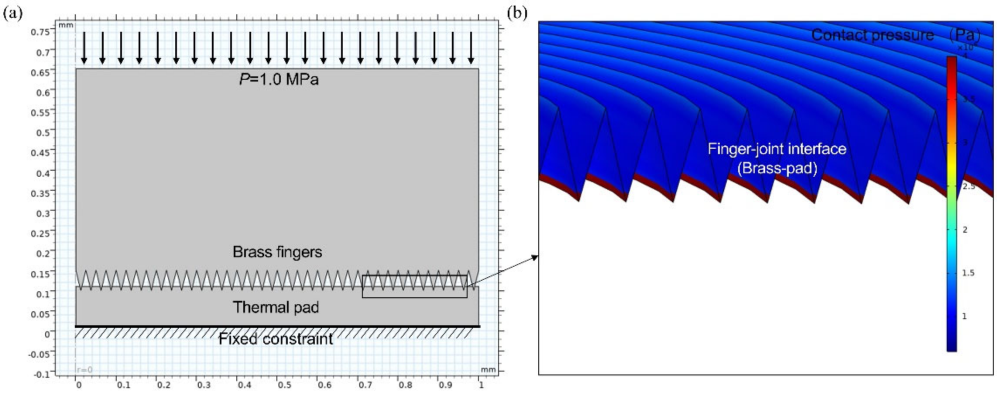

Figure S1. Axisymmetric finite-element verification of the force-amplification mechanism of the ideal finger array. (a) Model geometry and mechanical boundary conditions. The bottom surface of the thermal pad was fixed, and a uniform pressure of 1.0 MPa was applied to the upper surface of the brass substrate. (b) Contact-pressure distribution at the finger–pad interface.

The finite-element model was developed as an idealized mechanical verification of the geometric force-amplification mechanism rather than as a complete reproduction of the experimental brass–pad–brass assembly. The principal differences between the numerical and experimental systems are summarized in Table S1. In particular, the model considers a single finger–pad contact subjected to a uniform pressure, whereas the experiment contains two brass–pad

interfaces and is loaded through the complete reference-bar assembly. Accordingly, the numerical result is used to evaluate the local force-amplification mechanism and not to predict the absolute thermal resistance of the experimental system.

Table S1. Comparison of the boundary conditions and system settings used in the finite-element model and the experiment.

**Parameter or setting**	**Finite-element model**	**Experimental system**
Model configuration	Two-dimensional axisymmetric model of a single brass finger-array–pad contact	Complete double-sided brass–pad–brass assembly containing two finger-array–pad interfaces
Finger geometry	Ideal, regularly repeated triangular fingers with a nominal height of 50 μm and a pitch of 25 μm	Machined concentric ridges with finite peak and valley curvature; measured peak-to-valley height of approximately 35 μm
Applied loading	Uniform pressure of 1.0 MPa applied to the upper surface of the brass substrate	Axial load applied by calibrated weights to the complete assembly, corresponding to a nominal pressure of 1.0 MPa for the profile-characterization experiment
Upper mechanical boundary	Uniform distributed pressure	Load transmitted through the upper reference bar and upper brass substrate
Lower mechanical boundary	Bottom surface of the thermal pad fixed	Lower brass substrate supported by the lower reference bar and load-measurement assembly
Contact interfaces	One brass–pad contact pair	Two brass–pad contact interfaces
Contact formulation	Frictionless contact	Physical dry contact; the friction coefficient was not independently measured or prescribed
Material representation	Brass and thermal pad represented using the constitutive parameters specified in Section S1	Actual machined brass substrates and commercial thermal pad
Thermal conditions	Mechanical contact model only; no thermal boundary condition was applied	Steady-state heat flow through the complete brass–pad–brass assembly
Evaluated quantity	Scalar integral of the local normal contact pressure relative to the applied load	Area-normalized total and contact thermal resistances

Purpose	Verification of the ideal geometric force-amplification mechanism	Measurement of the thermal performance of the as-fabricated interface

1. Gent, A.N. On the relation between indentation hardness and Young's modulus. *Rubber Chem. Technol.* **1958**, *31*, 896–906. https://doi.org/10.5254/1.3542351
2. Henkel Corporation. BERGQUIST SIL PAD TSP 3500 Technical Data Sheet. Available online: https://datasheets.tdx.henkel.com/BERGQUIST-SIL-PAD-TSP-3500-en_GL.pdf (accessed on 26 September 2026).
3. Copper Development Association Inc. C36000 Alloy. Available online: https://alloys.copper.org/alloy/C36000 (accessed on 26 September 2026).
